# Novel *FOXL2* variants in two Chinese families with blepharophimosis, ptosis, and epicanthus inversus syndrome

**DOI:** 10.3389/fgene.2024.1343411

**Published:** 2024-02-12

**Authors:** Mingyu Zhao, Xiaolu Meng, Jiaqi Wang, Tailing Wang

**Affiliations:** ^1^ The Department of Facial and Neck Plastic Surgery, Plastic Surgery Hospital, Chinese Academy of Medical Sciences and Peking Union Medical College, Beijing, China; ^2^ Plastic Surgery Hospital, Chinese Academy of Medical Sciences and Peking Union Medical College, Beijing, China

**Keywords:** Blepharophimosis-ptosis-epicanthus inversus syndrome, FOXL2 variant, whole exome sequencing, tansfection, protein model prediction

## Abstract

**Introduction:** Blepharophimosis, ptosis, and epicanthus inversus syndrome (BPES) is a rare inherited disorder. This study was aimed to identify and functionally validate *FOXL2* variants in two Chinese families with BPES.

**Methods:** The proband and his family members were subjected to whole-exome sequencing to identify disease-associated variants. Several bioinformatic tools were used to computationally predict altered proteins. *In vitro* functional assays were conducted by transfecting wild-type and mutant *FOXL2* cDNAs into HEK-293 cells, followed by subcellular localization assays, luciferase reporter gene assays, and quantitative real-time polymerase chain reaction.

**Results:** The clinical features of BPES, including small palpebral fissures, ptosis, telecanthus, and epicanthus inversus, were present in all affected patients. Two novel mutations were detected, c.292T>A and c.383G>T. Whole-exome sequencing analysis and prediction software suggested that these mutations were pathogenic. Functional studies showed that these two point mutations decreased FOXL2 protein expression, resulting in subcellular mislocalization and aberrant transcriptional activity of the steroidogenic acute regulatory protein gene promoter.

**Conclusion:** Our results add to the current understanding of known *FOXL2* variants in, and our *in vitro* experiments provide reference data and insights into the etiology of BPES. Further studies are needed to identify the possible mechanisms underlying the action of this mutation on the development of BPES.

## 1 Introduction

Blepharophimosis, ptosis, and epicanthus inversus syndrome (BPES) is an autosomal dominant disorder with an incidence of 1 in 50,000 individuals. Two subtypes of BPES have been described: Type I is characterized by abnormal eyelid development and female infertility, whereas type II is characterized only by abnormal eyelid development and no infertility in either sex (De Baere et al., 2003; Nallathambi et al., 2008).

Approximately 70% of BPES cases are attributed to heterozygous variants of the *FOXL2* gene (De Baere et al., 2003; Kim and Bae, 2014; Bunyan and Thomas, 2019). Till date, over 270 *FOXL2* variants have been reported to be associated with BPES, 80% of which are intragenic mutations. These intragenic mutations are further categorized as shift (44%), in-frame (33%), nonsense (12%), and missense (11%) mutations. Although some defects are linked to genomic rearrangements involving *FOXL2*, most genetic defects in BPES result from intragenic mutations (Beysen et al., 2009). FOXL2 localizes to the nucleus and functions as a transcriptional regulator in the early development of eyelids and differentiation of ovaries, maintaining the genetic program. In addition, FOXL2 inhibits the components required for testicle development (Georges et al., 2014). Based on the clinical heterogeneity of patients with BPES, different mutations in *FOXL2* were suggested to be correlated with various BPES types. (De Baere et al., 2003; Fokstuen et al., 2003; Dipietromaria et al., 2009; Zhou et al., 2018). Therefore, identifying novel *FOXL2* variants and improving the understanding of the role of mutations in the pathogenesis of BPES may lead to the determination of biomarkers for early BPES detection and provide treatment targets for intervention. Thus, we aimed to identify and functionally validate *FOXL2* variants in two Chinese families with BPES.

Here, we identified and verified two *FOXL2* variants in two Chinese families with BPES. Functional studies of these two missense mutations, MT1 (c.292T>A) (Nallathambi, et al., 2008) and MT2 (c.383G>T), revealed significant alterations in both FOXL2 protein expression and the transcriptional repressive activity of the steroidogenic acute regulatory protein (StAR) promoter. Our findings emphasize the importance of these mutations in the etiology of BPES.

## 2 Materials and methods

### 2.1 Patients

Patients were recruited from the Department of Facial and Neck Surgery of our hospital. Whole-exome sequencing (WES) was performed by the Novogene Bio-informatics Co., Ltd. Beijing, China. The two families comprised a total of eight patients. Typical BPES manifestations, including palpebral fissures, telecanthus, and epicanthus inversus, were observed in three patients. Informed consent for participation in this study was obtained from all participants or their legal guardians in accordance with the tenets of the Declaration of Helsinki. Written informed consent was obtained from all participants, with parental consent secured for minors (those under 16). Written consent was also obtained for the use of photographs. This study was approved by the Ethics Committee of Plastic Surgery Hospital of the Chinese Academy of Medical Sciences (No. 2022-157).

### 2.2 Peripheral blood DNA extraction and WES

Peripheral blood (4 mL) was collected from each family member into EDTA-K2 anticoagulation tubes. Genomic DNA was extracted from leukocytes in the peripheral venous blood using a QIAamp DNA blood kit (Qiagen, Hilden, Germany). After obtaining the raw sequence reads, the data were analyzed by comparison with a reference sequence or genome (human_B37). The mutation nomenclature was in accordance with the guidelines of the Human Genome Variation Society. Mutations were identified using PolyPhen-2, SIFT (Sorting Intolerant from Tolerant), and MutationTaster.

### 2.3 Protein model prediction

The *FOXL2* gene coding sequence (NM_023067) was entered into AlphaFold2 to construct the model, which was imported into PyMOL (version 2.5; Schrödinger, LLC, New York, NY, United States). After importing the predictive model file, which was in the pdb format, into the software, the FOXL2-WT, p.Trp98Arg and p.Trp128Leu protein prediction models were constructed.

### 2.4 Plasmid Construction and Transfection

The coding sequence of *FOXL2* (NM_023067) was cloned into the pEGFP-N1 and pcDNA3.1-3xflag-N vectors. Mutant plasmids carrying c.292T>A and c.383G>T were generated using targeted mutagenesis polymerase chain reaction (PCR), using the wild-type (WT) FOXL2 expression vector as a template. The WT sequences were designated as pEGFP-WT and pcDNA-WT, and the two mutations as pEGFP-MT1 (c.292T>A), pEGFP-MT2 (c.383G>T), pcDNA-MT1 (c.292T>A) and pcDNA-MT2 (c.383G>T) respectively. The human StAR promoter was constructed using pGL3-basic (Promega, Madison, WI, United States) as the luciferase reporter vector. All constructs were sequenced and validated by Sanger sequencing.

Human embryonic kidney (HEK)293T cells were obtained from our laboratory stock. The cells were cultured in complete medium consisting of Dulbecco’s modified Eagle’s medium (DMEM; Gibco™, United States) with 10% fetal bovine serum (Gibco™) at 37°C and 5% CO_2_. Lipofectamine 2000 (Invitrogen, Carlsbad, CA, United States) was used for transfection. The pEGFP-N1 and pcDNA3.1-3xflag-N plasmids were gently mixed in 250 μl Opti-MEM™ I Reduced Serum Medium (Gibco™, United States). Lipo-2000 (10 µL) was gently mixed into 250 µL Opti-MEM™ I Reduced Serum Medium and incubated for 5 min at 20°C–25 °C. The diluted DNA mixture was combined with the Lipo-2000 mixture and incubated for 20 min at 20°C–25°C. After 4–6 h, the medium was replaced with complete medium.

### 2.5 Luciferase reporter gene assay

We used 24-well plates for transcriptional activity assays. HEK293T cells were transfected with the empty vector pcDNA3.1-3xflag, the WT or mutant *FOXL2* expression vector, and the above-mentioned reporter gene constructs using Lipofectamine 2000 reagent (Invitrogen). Four groups were co-transfected into HEK293 cells, each containing 500 ng of either pcDNA3.1-3xflag, pcDNA-WT, pcDNA-MT1, or pcDNA-MT2, in addition to 500 ng of luciferase reporter plasmid (pGL3-StAR) and 40 ng of pRL-TK plasmid (Promega). The total DNA content of each well was maintained at 1,040 ng/well. Cells were incubated with plasmid in DMEM for 8 h and then in complete medium for 48 h. Luciferase intensity was measured using an EnSpire Multiplex Plate Reader (PerkinElmer, Waltham, MA, United States) with a dual-luciferase reporter gene assay system (Promega). All experiments were performed in triplicate.

### 2.6 Subcellular localization assays

HEK293T cells were transfected with Lipo 2000 and plasmids containing the empty vectors pEGFP-N1, pEGFP-FOXL2, pEGFP-FOXL2-MT1, and pEGFP-FOXL2-MT2(Plasmid and Lipo-2000 were used at the dosages described in the Plasmid Construction and Transfection section). At 24 h after transfection, cell nuclei were re-stained with Hoechst 33,342 (Beyotime Institute of Biotechnology, Jiangsu, China) and observed under a fluorescence microscope (Olympus, Tokyo, Japan).

### 2.7 Real-time PCR

To assess whether the mutations affected the expression of the downstream target gene *StAR*, SYBR Green real-time quantitative (q) PCR was performed using LightCycler^®^ 96 Instrument (Roche, Basel, Switzerland). The pcDNA3.1-3xflag expression vector (4 µg) containing WT or mutant *FOXL2* cDNA and empty pcDNA3.1-3xflag vector were transfected into HEK293T cells in 12-well plates using Lipo 2000. After 48 h, total mRNA was extracted from the cells using TRIzol (Invitrogen). mRNA was reverse transcribed into cDNA using a Reverse Transcription Kit (Takara). The cDNA (2 μg) was subjected to real-time PCR using FastStart Universal SYBR Green Master (Rox). *GAPDH* was used as an endogenous control to standardize data. The following primers were used *StAR*-F: 5′-CAG​ACT​TCG​GGA​ACA​TGC​CT-3′; *StAR*-R: 5′-CCC​TTG​AGG​TCG​ATG​CTG​AG-3′; *GAPDH*-F: 5′-CTG​CCA​ACG​TGT​CAG​TGG​TG-3′; and *GAPDH*-R: 5′-TCA​GTG​TAG​CCC​AGG​ATG​CC-3′.

## 3 Results

### 3.1 Gene analysis and protein model construction

**FIGURE 1:**
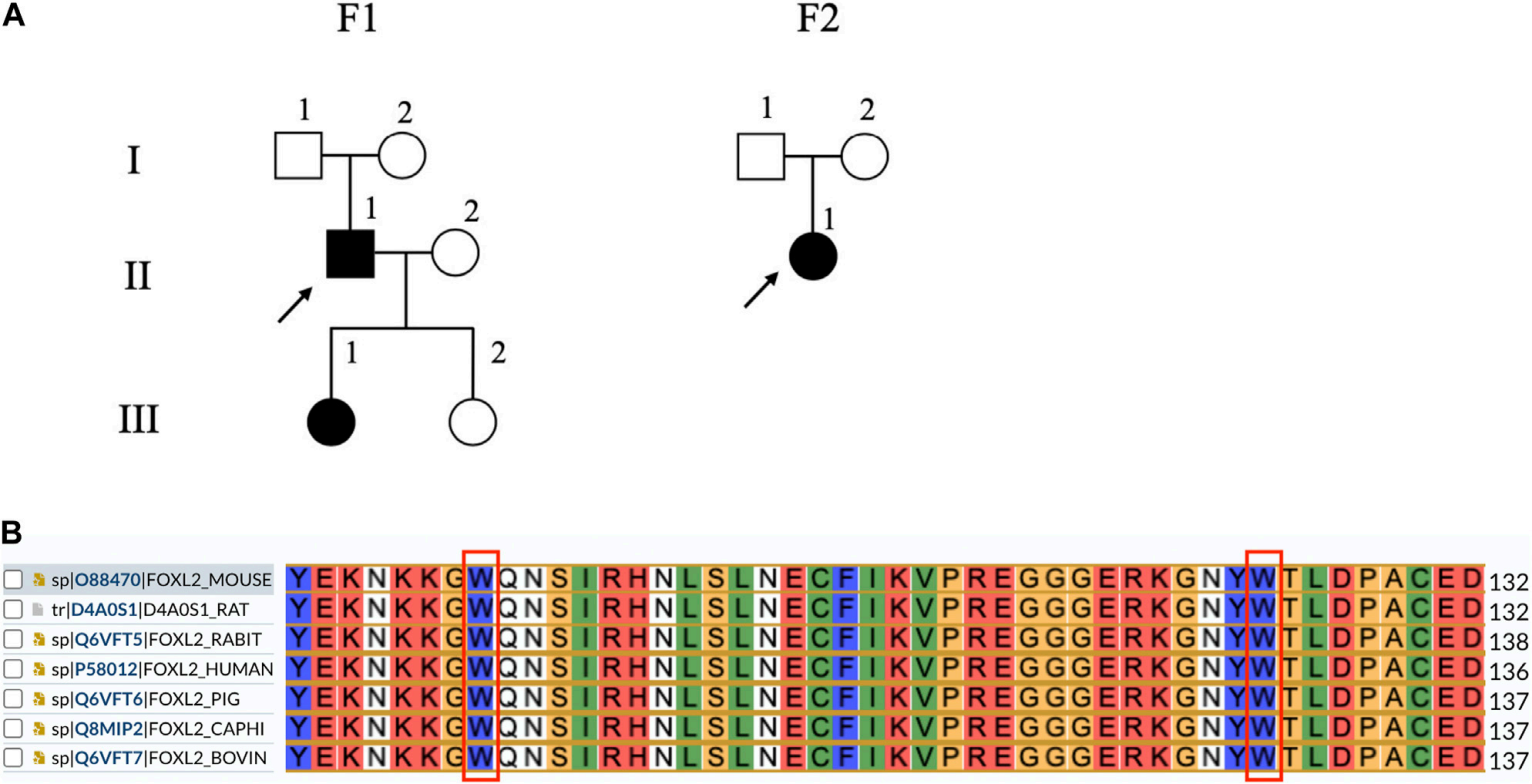
Lineage and genomic analysis of *FOXL2* and sequence alignment of seven *FOXL2* homologs. **(A)** Affected individuals are indicated by filled symbols and preceding witnesses are marked with upward arrows. **(B)** Comparison of *FOXL2* protein sequence around 98 and 128 by using UniProt indicates that the residues surrounding these two mutations are significantly conserved.

The results of WES indicated the presence of the c.292T>A mutation in families III(1) and II(1) in F-1 and that of the c.383G>T mutation in family II(1) in F-2. The results of the polyPhen-2, SIFT, and MutationTaster programs revealed that the mutations were situated in the forkhead structural domain and that their positions are widely conserved in mammals (Figure 1B). Both mutant amino acids were predicted to damage the forkhead protein (Table 1).

**TABLE 1:** Prediction results of PolyPhen-2, SIFT and MutationTaster programs.

Mutation	SIFT and score	Polyphen2 and score	MutationTaster and score
c.292T>A	Deleterious, 0.001	Probably damaging, 0.995	Disease causing, 1
c.383G>T	Deleterious, 0.001	Probably damaging, 0.988	Disease causing, 1

**FIGURE 2:**
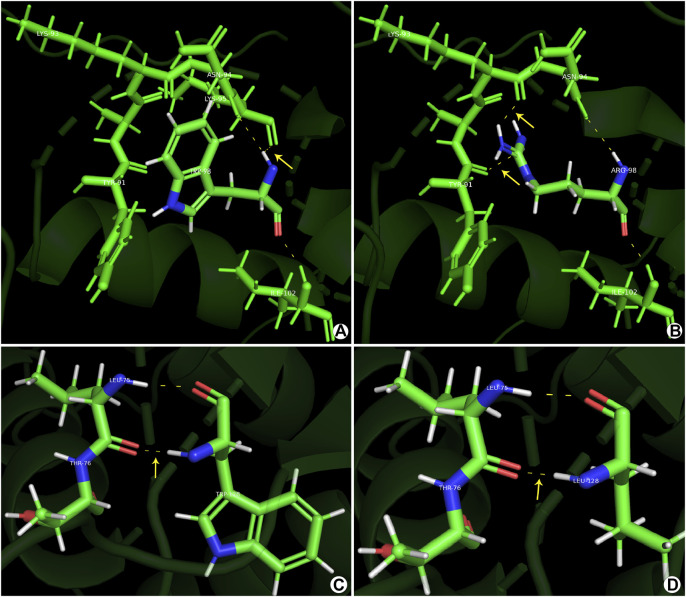
Prediction models constructed using the PyMOL software. **(A, C)** Normal gene conformation. **(B)** MT1 (p.Trp98Arg) shows a novel hydrogen bond between ARG-98 and TYR-91. **(D)** MT2 (p.Trp128Leu) shows stable primary conformation but altered secondary structure with the amino acid changes to leucine.

Protein structure analysis showed that for c.292T>A(p.Trp98Arg), the tryptophan isopotential was 5.89. The mutation of tryptophan (a non-polar amino acid) to arginine (a basic amino acid) resulted in an isopotential of 10.76; thus, the mutation increased the potential difference between amino acids. Simultaneously, the hydrogen bonds between the amino acids increased, resulting in a change in the protein structure and alterations in the protein function. c.383G>T(p.Trp128Leu) has a leucine isopotential of 6.01 compared to the WT. Although the potential difference remained mostly unchanged, changes in the local spatial structure of the forkhead structural domain were observed (Figure 2). Thus, mutant amino acids may have important effects on polarity, such as disrupting protein–DNA interactions.

### 3.2 Subcellular localization

**FIGURE 3:**
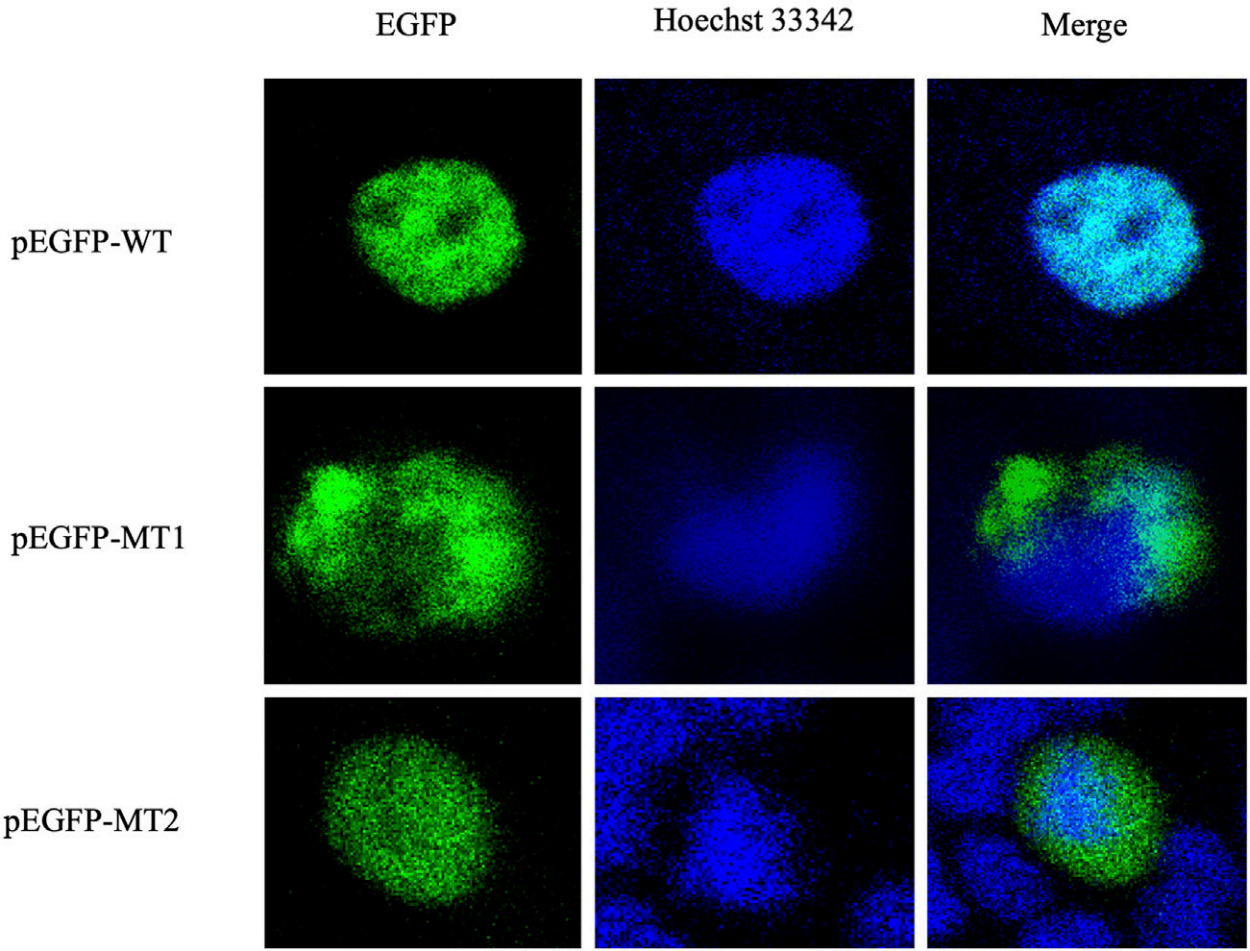
Protein expression and distribution. Subcellular localization of EGFP, FOXL2, FOXL2-MT1, and FOXL2-MT2. The first column shows the subcellular localisation of EGFP as a marker for FOXL2 protein; the second column shows the nuclei stained with Hoechst33342. The third column shows the combined images of the above images (400× magnification).

Localization studies in HEK293T cells revealed the effect of the mutations (c.292T>A and c.383G>T) on the subcellular localization of FOXL2. As shown in Figure 3, WT FOXL2 was localized to the nucleus, which is consistent with its function as a transcription factor. In contrast, cells transfected with mutant constructs (c.292T>A and c.383G>T) showed a non-WT distribution (Figure 3).

### 3.3 Dual luciferase reporter gene assay

**FIGURE 4:**
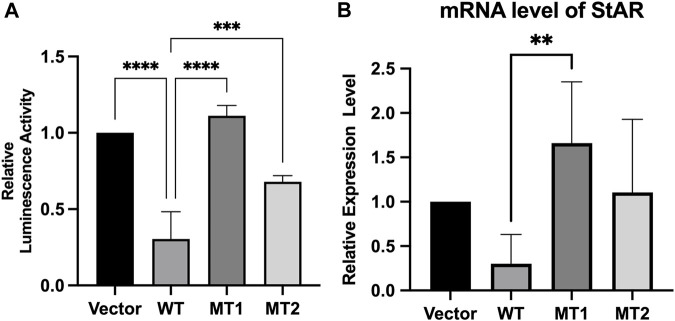
Transcriptional activity of c.292T>A mutant *FOXL2* and c.383G>T mutant *FOXL2*. **(A)** The plasmids pcDNA3.1 (vector), pcDNA3.1 - *FOXL2* (WT), pcDNA3.1 - *FOXL2* - (MT1) or *FOXL2* - (MT2) were co-transfected with the luciferase vectors driven by the *StAR* promoter. **(B)** The relative expression level of *StAR*, as measured by RT-PCR, was compared between the cells transfected with empty vectors and those transfected with WT and MT plasmids. ****p < 0.01, **p < 0.05.

We assessed the ability of mutant constructs to transactivate the *StAR* promoter, a target of FOXL2 (Zhou et al., 2018; Li et al., 2021) using luciferase-based reporter gene assays. The results indicated that WT *FOXL2* inhibited *StAR* promoter activity, which was not significantly inhibited in cells transfected with equivalent amounts of mutant *FOXL2* constructs (c.292T>A and c.383G>T) (*p* < 0.05). These results indicate that *FOXL2* with c.292T>A and c.383G>T mutations lost its function (Figure 4A).

### 3.4 Real-time qPCR

We further evaluated the effect of the novel *FOXL2* variants (c.292T>A and c.383G>T) on transactivation capacity of *StAR* by measuring the endogenous *StAR* mRNA expression using real-time PCR. *StAR* mRNA expression was significantly higher in cells transfected with c.292T>A and c.383G>T than that in WT-*FOXL2* cells. These results are consistent with those of the dual luciferase reporter gene assay (Figure 4B).

## 4 Discussion

FOXL2 is an evolutionarily highly conserved transcription factor that was first identified as a BPES susceptibility gene. It plays important roles in sex determination, reproduction, metabolism, and tumor formation. It has also been implicated in the pathogenesis of several diseases, including polycystic ovarian syndrome, keloids, and reproductive tumors (Nakashima et al., 2010). Mutations in a single *FOXL2* allele result in reduced levels of functional *FOXL2* expression, and mutations in two *FOXL2* alleles can be lethal (Chai et al., 2017). Thus, patients with BPES typically have heterozygous mutations.

As reported previously, nearly 90% of patients with BPES have genetic defects involving *FOXL2* (Verdin and De Baere, 2012). In the present study, we identified and characterized two missense mutations, MT1 and MT2, which are located in the forkhead structural domain and alter evolutionarily conserved amino acids. The spatial conformation of the protein was altered, and the properties of the amino acids at the mutated sites were changed, resulting in impaired protein function. Both mutations were predicted to be deleterious. The subcellular localization results indicated that the mutations led to gene expression in the cytoplasm outside the nucleus. These results strongly suggest that the two novel mutations, MT1 and MT2, are pathogenic and contributed to the pathogenesis of BPES in our patients.

MT1 and MT2 were less capable of downstream promoter activation than the WT, confirming that the c.292T>A and c.383G>T mutants caused loss of function of the *FOXL2* gene. This may have occurred because of decreased levels of FOXL2 in the nucleus, where the mutation reduced the rate of FOXL2 translocation. In addition, soluble molecules with misfolded proteins may not recognize the binding sites in the target promoter. c.292T>A and c.383G>T attenuated the transactivation of downstream StAR, possibly because of the subcellular mislocalization of mutant *FOXL2*, loss-of-function of the mutant protein, or other reasons related to downstream gene activation. This was consistent with the results of the previously discovered mutant c.383G > A (Li, et al., 2021). Additionally, eyelid development is highly sensitive to changes in the amount of *FOXL2* (Yan et al., 2023). Based on the above evidence, *FOXL2* variants may contribute to developmental malformations of the eyelid.

In conclusion, we report two pathogenic *FOXL2* variants (c.292T>A and c.383G>T) and confirm for the first time that both missense mutations lead to reduced expression and activity of *FOXL2* protein. This study extends the range of known *FOXL2* variants and contributes to the understanding of the etiology of BPES.

## Data Availability

The data uploaded to the SRA is open access at https://www.ncbi.nlm.nih.gov/sra/. The numbers are: SRR27500370, SRR27500369, SRR27500368, SRR27500367, SRR27407349, SRR27407348, SRR27319410, SRR27319409, SRR27319408, SRR27319407.
