# Corrigendum: Novel FOXL2 variants in two Chinese families with blepharophimosis, ptosis, and epicanthus inversus syndrome

**DOI:** 10.3389/fgene.2024.1414939

**Published:** 2024-04-29

**Authors:** Mingyu Zhao, Xiaolu Meng, Jiaqi Wang, Tailing Wang

**Affiliations:** ^1^ The Department of Facial and Neck Plastic Surgery, Plastic Surgery Hospital, Chinese Academy of Medical Sciences and Peking Union Medical College, Beijing, China; ^2^ Plastic Surgery Hospital, Chinese Academy of Medical Sciences and Peking Union Medical College, Beijing, China

**Keywords:** blepharophimosis-ptosis-epicanthus inversus syndrome, FOXL2 variant, whole exome sequencing, tansfection, protein model prediction

In the published article, there was an error in the legend for **Figure 3** as published. The text of the legend for **Figure 3** is written backwards: “Protein expression and distribution. Subcellular localization of EGFP, FOXL2, FOXL2-MT1, and FOXL2-MT2. The first column shows the nuclei stained with Hoechst33342; the second column shows the subcellular localization of EGFP or FOXL2 as an EGFP-tagged fusion protein. The third column shows the combined images of the above images (400× magnification).”

The contents of the first and second columns should be exchanged. The corrected legend should read as “The first column shows the subcellular localisation of EGFP as a marker for FOXL2 protein; the second column shows the nuclei stained with Hoechst33342.”

The authors apologize for this error and state that this does not change the scientific conclusions of the article in any way. The original article has been updated.

